# Surgical workflow simulation for the design and assessment of operating room setups in orthopedic surgery

**DOI:** 10.1186/s12911-020-1086-3

**Published:** 2020-07-02

**Authors:** Juliane Neumann, Christine Angrick, Celina Höhn, Dirk Zajonz, Mohamed Ghanem, Andreas Roth, Thomas Neumuth

**Affiliations:** 1 Innovation Center Computer Assisted Surgery (ICCAS), Leipzig University, Semmelweisstr. 14, 04103 Leipzig, Germany; 2 Department of Orthopaedic, Trauma and Plastic Surgery, Division of Endoprothetic Joint Surgery and General Orthopaedics, University of Leipzig Medical Center, Leipzig, Germany

**Keywords:** Surgical workflow simulation, Discrete event simulation, Operating room management, Surgical process optimization

## Abstract

**Background:**

The design and internal layout of modern operating rooms (OR) are influencing the surgical team’s collaboration and communication, ergonomics, as well as intraoperative hygiene substantially. Yet, there is no objective method for the assessment and design of operating room setups for different surgical disciplines and intervention types available. The aim of this work is to establish an improved OR setup for common procedures in arthroplasty.

**Methods:**

With the help of computer simulation, a method for the design and assessment of enhanced OR setups was developed. New OR setups were designed, analyzed in a computer simulation environment and evaluated in the actual intraoperative setting. Thereby, a 3D graphical simulation representation enabled the strong involvement of clinical stakeholders in all phases of the design and decision-making process of the new setup alternatives.

**Results:**

The implementation of improved OR setups reduces the instrument handover time between the surgeon and the scrub nurse, the travel paths of the OR team as well as shortens the procedure duration. Additionally, the ergonomics of the OR staff were improved.

**Conclusion:**

The developed simulation method was evaluated in the actual intraoperative setting and proved its benefit for the design and optimization of OR setups for different surgical intervention types. As a clinical result, enhanced setups for total knee arthroplasty and total hip arthroplasty surgeries were established in daily clinical routine and the OR efficiency was improved.

## Background

In the last decades, many efforts were put into the improvement of the operating room (OR) design and layout efficiency [1, 2]. Since the nineteenth century, the OR design has changed from anatomy theatres with visitor galleries to a highly aseptic and technical environment [2]. The integration of large-scale medical devices (e.g. intraoperative MRI or X-ray devices) and innovative surgical methods (e.g. minimal invasive and robotic surgery) in the OR have led to an increase of the operating room size in recent years [3]. As the ORs become larger, also the spatial distances in the OR and between functional units such as supply areas, sterilization as well as anesthesia and post-anesthesia care units increase. This results in ineffective traffic patterns and long travel paths for the OR staff [1]. The OR design and surgical department layout have an essential impact on the intraoperative processes, and therefore the overall efficiency of the surgical procedures. For example, recent studies showed the correlation between OR and equipment layout and surgical workflow disruptions [4, 5].

The optimal layout of surgical departments has been widely analyzed from different perspectives, e.g. patient flow [6, 7], efficiency and economics [8–11], hygiene [12], patient outcome [13] and equipment [14]. Nevertheless, little attention has been devoted so far to the design of the internal OR layout with respect to OR and instrument tables as well as staff positions. Attempts to determine the optimal design for special interventions, such as hybrid ORs [3, 15] and endoscopic surgery suites [16] were reported. However, there is no method for the assessment and design of operating room setups for different surgical disciplines and intervention types available. Although, the OR setup is influencing the team’s collaboration and communication [17], ergonomics, as well as intraoperative hygiene substantially, the table layout is set up mostly based on the subjective preference of the lead surgeon or based on institutional practices.

The aim of this work is to establish an optimal OR setup for common procedures of total joint arthroplasty. Thereby, the collaborative surgical processes, ergonomics and physical positions of the surgical team in the operating room, positions and setups of instrument tables as well as travel paths in the OR were considered. With the help of computer simulation, a method for the design and assessment of improved OR setups was developed. During development, special attention was given to the integration of clinical stakeholders in all phases of the design, decision-making and evaluation process. The resulting methodology enables a comparison of different OR setup alternatives, which could lead to improved intraoperative processes and reduced the procedure duration. Subsequently, the best-performing OR setups were intraoperatively evaluated regarding their impact on the surgical processes and overall efficiency of the procedure.

### State of the art

Currently, the internal OR layout is set up mostly based on the subjective preference of the lead surgeon, the scrub nurse or institutional guidelines. In the orthopedic department, every scrub nurse or surgeon had their own preferences for instrument table layout, which led to a variety of different setups. An objective assessment method was needed in order to compare the OR setup performance from different perspectives.

Computer simulation has been demonstrated to be useful in the clinical decision-making process and systems improvement by identifying process optimization potential and efficiency gains [18, 19]. In literature, different simulation techniques, such as Monte Carlo simulation, discrete event simulation, system dynamics, and agent-based simulation have been applied to a variety of different problems in healthcare applications [20]. Hybrid simulation combines two or more of those techniques and has been applied to the healthcare domain in recent research [21–23]. Computer simulation has been widely used for the representation, analysis, optimization, and prediction of hospital and OR processes. In hospital management, simulation has been applied for the analysis and optimization of pathway and workflow planning [24], optimization of patient flow [25] and resource planning [26]. In the OR setting, simulation methods have been also utilized, mainly to improve the OR scheduling [27–31] and the patient flow [7, 32, 33]. A detailed review of OR scheduling and planning using DES and other simulation techniques as well as mathematical models can be found in [34].

In this work, Discrete Event Simulation (DES) was utilized for the design and assessment of operating room setups. DES is a methodology to (re-) design, analyze, execute and evaluate processes in respect of different situations or objectives. DES enables the emulation and prediction of changes in a dynamic model of a real-world system over time via mathematical modeling [32]. The simulation allows a safe, repeatable analysis of a situation and the impact of different parameters and process configurations on the process, e.g. methods and strategies, process alternatives, different activity durations or availability of personnel and material resources [35–37]. DES models the behavior of a system as a discrete sequence of events. Each event triggers a state change in the system. During DES only consecutive events are simulated. Between those events, no system behavior is assumed. This enables the simulation of long periods in a shorter time, which is beneficial especially in healthcare applications.

DES is the method most used for the simulation of operating room workflows [6, 38]. For example, in the domain of intraoperative process optimization, Fernández-Gutiérrez et al. used DES methods in order to find the optimal development of new complex procedures in multimodal imaging environments [39] and for resource optimization of medical equipment [40]. Khoshkenar et al. used DES to model the traffic flow in the OR for the improvement of the OR layout based on the distance walked by the OR staff [41].

Analyzing success stories in healthcare simulation, stakeholder engagement has been identified as a critical factor for the realization of simulation and optimization projects [19, 42]. The project success depends on the engagement of those who are affected by changes in the process [43]. The integration of different stakeholders’ knowledge and experiences in simulation projects enables a shared view of the problem from multiple perspectives as well as a collaborative decision-making process [42]. Jahangirian et al. identified the most critical factors for low clinical stakeholder integration: organizational factors (e.g. high workload of clinicians, communication gap between simulation experts and clinical stakeholders), technical factors (e.g. difficulties in working with simulation methods and tools), project management factors (e.g. extensive length of projects, poor team efforts) and healthcare-specific factors (e.g. more complex problems) [19]. Healthcare simulation projects have been identified as more difficult then simulation projects in other industry domains [44]. According to [44], healthcare simulation often struggles with more complex systems and less evident structure, messier problems, less client time and less appropriate simulation software. Additionally, it is more difficult to access and collect relevant data for simulation modeling and implementation. In the operating room, all stakeholders form one team, which leads to multiple decision-makers and the need to find a common consensus for realizing a successful simulation project as well as implementing the results in daily clinical routine.

In literature, a stakeholder involvement plan is suggested for collaborative user engagement, by conducting workshops, surveys, and stakeholder meetings on a regular basis [18, 42]. Additionally, visual representations of the operating room have been shown to be effective, for the involvement of clinical stakeholders in the development process. User involvement enables a consensus-based decision-making process and improves the design of prototypes [45]. There are different techniques available, such as full-scale mockups and simulation [46] or virtual reality environments [47]. In this study, discrete event simulation, which provides a highly visual 3D model of the operating room was used for the improvement of the internal OR layout and to engage user involvement in the design process. In addition, the mathematical modeling functionalities of DES supports the analysis of the intraoperative process from different perspectives.

To the best of our knowledge, DES methods have not been utilized yet for the design of enhanced OR setups considering intraoperative processes, instrument table positions, OR staff ergonomics, and the travel path of the circulator. In this study, DES enables a quantifiable comparison of different setup options based on defined simulation parameters (e.g. handover time, travel paths). Additionally, a 3D graphical representation of the simulation software provided an adequate environment for the involvement of clinical stakeholders in the design of the new setup alternatives and the group-based decision-making process.

## Methods

**Fig. 1:**
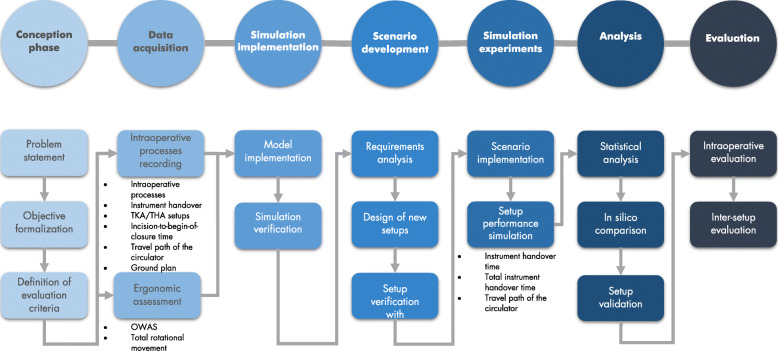
Methodology for the design and assessment of OR setups (adapted from Law and Kelton [35])

For the development of improved OR setups, a methodology was designed and is presented in Fig. 1. The methodology was adapted from the basic structure of simulation studies presented by Law and Kelton [35]. The methodology integrates clinical stakeholders in all phases of simulation development, implementation, and evaluation. After every major work step, results were presented in stakeholder workshops and user surveys were conducted. The discussion results were then considered in the next steps of simulation development.

Firstly, the problem of inefficient OR setups was identified, and the study objectives have been formalized in cooperation with the clinical stakeholders. Additionally, the two most common arthroplasty procedures were identified as adequate use cases and evaluation criteria of the study have been defined in user interviews (Conception phase). For the assessment of the operating room setups, various intraoperative data were acquired, and an ergonomic assessment of the surgical team was performed (Data acquisition). With the help of DES software, a 3D simulation model of the orthopedic OR was implemented and verified with the intraoperatively recorded data (Simulation implementation). Based on the initial simulation and the as-is analysis, requirements for an optimal OR setup were defined in stakeholder workshops, and new OR setups for orthopedic surgery were designed (Scenario development). In the next step, the newly designed setups were implemented in the simulation environment (Simulation experiments) and simulation experiments have been performed. Then the simulation results were analyzed from different perspectives and the improved setups have been clinically validated (Analysis). Finally, the improved setups were evaluated in the actual intraoperative setting (Evaluation).

### Conception phase

#### Problem statement

Initially, the procedural and structural problems in the orthopedic OR at the University of Leipzig Medical Center, Division of Endoprothetic Joint Surgery and General Orthopaedics, were identified in discussion with the relevant stakeholders. The surgeons and nursing staff reported a physical strain during surgeries due to static positions with bent or twisted back and head as well as prolonged standing. The scrub nurses, who are responsible for the handover of sterile items to the surgeon, stated insufficient instrument handovers during the surgery. The circulators, who provide assistance to the OR team (e.g. brings and handle aseptic supplies), described structural problems within the OR layout and long travel paths between material stocks.

#### Objective formalization

To address these problems, the study aimed at the improvement of the OR setups and the implementation of a standardized setup for two common orthopedic surgeries at the University of Leipzig Medical Center. The main objective of this study was to improve the overall efficiency of the surgical procedure by optimizing the OR setups for Total Hip Arthroplasty (THA) and Total Knee Arthroplasty (TKA) surgeries. THA and TKA surgeries were defined as a use case within this study. In THA surgeries, the hip joint is replaced by a prosthetic implant to treat arthritis pain or hip fractures. With over 1.4 million cases per year [48], it is one of the most performed surgeries worldwide. During TKA the knee joint is replaced to relieve debilitating pain or osteoarthritis. With over 1.1 million cases per year worldwide, TKA surgery is also a commonly performed orthopedic procedure [49]. Although surgical implants surgeries bear a high risk for surgical site infections, the infection rates are less common than e.g. catheter-related infections [50]. Nevertheless, the treatment of implant infections is complex, longer and more cost-intensive [50, 51]. Therefore, intraoperative hygiene for THA and TKA surgeries is in continuous consideration and optimization. Besides hygienic aspects, also economic and ergonomic considerations are highly relevant during OR layout design for THA and TKA surgeries.

#### Evaluation criteria

According to the identified problems, special attention was given to increase instrument handover efficiency between the scrub nurse and the surgeon as well as optimize the travel path of the circulator. In addition, the surgery duration should be reduced, and the surgical teams’ ergonomic situation should be improved. For this purpose, different simulation scenarios were developed, implemented and analyzed. The evaluation of the OR setup improvements was performed in the intraoperative setting.

### Data acquisition

#### Intraoperative process recording

Initially, 15 THA and 7 TKA surgeries were recorded at the University of Leipzig Medical Center, Division of Endoprothetic Joint Surgery and General Orthopaedics in 2016 for the intraoperative process optimization and setup improvement. The results of the data acquisition are presented in Results.

During process acquisition, the intraoperative processes for THA and TKA were manually recorded with a low granularity level. Thereby, the duration of the surgery (incision-to-begin-of-closure time (IBCT)), as well as the used OR setups for THA and TKA with positions of instrument tables and the position of the OR staff, were acquired. For every OR setup the amount, duration and pathway of instrument hand-over between the surgeon and scrub nurse were measured. The organization of instruments on the table is not considered in this study. Furthermore, the pathways of the circulator were recorded for every THA and TKA OR setup.

The ground plan of the orthopedic OR at the University of Leipzig Medical Center was digitized for the modeling in the simulation environment. Additionally, dimensions of furnishing, OR table (fixed position in the OR), instrument tables (mayo stands, instrument/equipment stands, solutions stands, etc.), medical devices (e.g. c-arm and anesthesia equipment) as well as OR display were acquired. The dimensions were integrated into the 3D simulation model to create a realistic image of the orthopedic OR.

#### Ergonomic assessment

##### Ovako working posture analyzing system

The ergonomic assessment of the initial OR setups for THA and TKA was performed using the *Ovako Working Posture Analyzing System* (OWAS). The method was developed by Karhu et al. in 1977 [52] and is used to ergonomically classify working postures of the back, arms, legs and optionally the head. For the ergonomic evaluation of the operating room theatres, OWAS was already used for different surgical disciplines [48, 53, 54] as well as different occupational groups, like surgeons [55] and scrub nurses [56].

Especially, orthopedic surgeons have an increased risk of musculoskeletal injuries and disorders due to the more physical demands of the surgeries [57, 58]. To address this problem, the intraoperative situation in the orthopedic OR should be improved. Therefore, the OWAS assessment was performed for the surgeon, assistants, scrub nurse and the circulator in THA and TKA surgery for the initial OR setups. A detailed analysis of the ergonomic situation in the orthopedic OR as well as all results of the OWAS assessment can be found in [59]. The evaluation results showed ergonomic critical positions most of the time for the surgeon, the scrub nurse and the assistants regarding bent or twisted back and head postures as well as static positions and prolonged standing during surgery. Based on the findings of the OWAS assessment, requirements for ergonomically improved OR setups were defined.

##### Total rotational movement (TRM)

In addition to the OWAS assessment, the amount of rotational movement, which is performed by the surgeon and the scrub nurse during an instrument handover, was calculated with a simplified theoretical model. The body rotation depends significantly on the OR setup and has a great impact on the staffs’ ergonomics. The theoretical degree of rotation was calculated for each OR setup by determining and adding the angles between the tables and persons for an instrument handover. For simplicity, it has been assumed that the scrub nurse rotates the torso according to the instrument tables’ angle. The *Total Rotational Movement of an OR table TRM*_*Table*_ is defined as

$$ {TRM}_{Table}=\left[\left(i\cdot \alpha \right)+\left(j\cdot \beta \right)+\left(k\cdot \gamma \right)\right]\kern0.48em with\kern0.24em i,j,k\kern0.28em \in \kern0.28em \mathbb{N}\kern0.28em and\kern0.28em 0\le i,j,k\le 2 $$

**Fig. 2:**
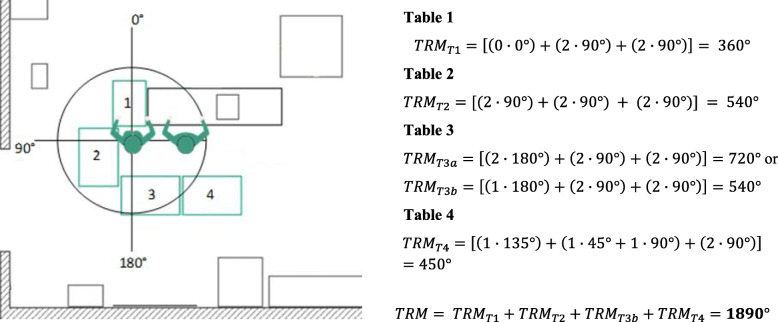
Example of the simplified theoretical model to calculate the Total Rotational Movement of an OR setup, including the scrub nurse (left) and surgeon (right)

α may be the angle of rotational movement of the scrub nurse for grabbing an instrument from an instrument table. β is defined as the rotation angle of the scrub nurse for giving the instrument to the surgeon and γ is the angle of the surgeons’ rotational movement for grabbing the instrument from the scrub nurse. Let i, j, and k be the number of movements for one rotational movement. In most cases, the person needs to move two times – firstly, to the target (table or person) and secondly back in the original position. The Total Rotational Movement of the OR setup is the sum of the overall TRM_Table_. In Fig. 2 an example of the rotational model for a left-side THA OR setup is presented.

### Simulation implementation

To create a realistic digital OR simulation environment for the evaluation of different OR setups, a simulation environment was utilized. Following the argumentation in [40], Delmia by Dassault Systèms [60] was used to implement THA and TKA intraoperative processes. A description of the Delmia simulation environment and a detailed representation of the simulation implementation can be found in Appendix A.

### Scenario development

#### Requirements analysis

The positioning of the OR table, instrument tables, and the OR personnel is mostly defined by the preference of the main surgeon or the scrub nurse. Even for the same intervention type in the same institution, there are often different OR setups implemented. The OR layout is affecting the surgical processes, the staffs ergonomic as well as team collaboration in the OR. For example, the impact of instrument table positions on the surgeon and scrub nurse instrument transfer performance was analyzed in [17]. The study showed that the alignment (gaze direction) and the relative position of the table in the proximity of the surgeon have an influence on the handover time [17].

However, current research lacks in the objective determination of how an efficient and well-organized OR setup could be defined for different intervention types and surgical disciplines and which aspects need to be considered in the design phase. Due to the lack of literature, a non-exhaustive list of requirements was identified with the help of ergonomic assessment, process analysis, and clinical expert interviews. For this purpose, 22 orthopedic procedures were observed with respect to their functional, spatial and ergonomic requirements. Additionally, a brain-storming workshop with 3 experienced senior surgeons and 5 scrub nurses was conducted to define the requirements of an optimal OR setup. The identified aspects are presented in Table 1 and should be considered in the design of OR setups.

**Table 1:** Requirements considered during the intraoperative design of optimal OR design

***Functional requirements***		
1	Alignment of instrument tables	For the design of optimal OR setups, the positioning of instrument tables should allow the whole OR team, and especially the surgeon, 1st assistant and scrub nurse a direct view on the operating area whenever possible. Hence, the scrub nurse is able to anticipate the surgeons’ actual and next needs (e.g. instrument or material handover), which has a positive impact on the process flow of the surgery [17].
2	Relative positioning of instrument tables in proximity to the surgeon	The instrument tables should be positioned in adequate proximity to the surgeon. This shortens the paths for instrument handover between the scrub nurse and the surgeon [17].
***Spatial requirements***		
3	Freedom of movement	The space for the OR staff, especially for the surgeon, should be planned generously to ensure unrestricted freedom of movement during surgery. There should also be sufficient space available in the setup if large-sized equipment is used during surgery [14].
4	Planning of pathways	The pathways to supply stocks, workstations and the sterile area should be as short as possible to minimize the travel path of the circulator during surgery [61].
5	Sufficient space for medical devices	If large-sized medical devices (e.g. c-arm or a surgical microscope) are needed during surgery, sufficient space should be planned in the setup. When the equipment is not in use, it should be located opposite to the surgeon and in proximity to the operating area to enable a fast preparation and set up.
***Hygienic requirements***		
6	Positioning of the instrument tables next to or in front of the surgeon	The instrument tables should not be located behind the surgeon. Although in most cases the surgeon is dressed completely in sterile clothing, the back is considered as less sterile due to the clothes closure.
7	Minimizing staff circulation	The pathways of the OR staff, especially the circulator, should not impinge on the instrument tables. The airflow and unintentional contacts may cause physical and bacteriological effects and increases the risk of instrument and implant contamination.
8	Positioning of the instrument tables in the proximity of the OR table	The instrument tables should be positioned in proximity of the OR table, which is specially protected against pathogens by a sterile area with a stable flow of filtered air.
***Ergonomic requirements***		
9	Minimizing the rotational movement of the OR staff	Where possible the instrument tables should be positioned next to or in front of the surgeon and in a U-shape in front of the scrub nurse. This minimizes rotational movement and improves an ergonomic body posture.
10	Avoiding twisted or bent body postures	The height of the OR table and instrument tables should be adapted to the individual needs of the OR personnel [62]. In addition, the operating area and displays in the OR should be seen by the personnel without bending or twisting the head or back.

#### Design and verification of new OR setups for THA and TKA

Considering these requirements as well as the results of the OWAS and TRM assessment, improved OR layouts for THA and TKA surgeries were designed in the 3D environment of Delmia. In addition to the initial setups, three new TKA setups and one new THA Setup were selected for further analysis, simulation, and comparison. The newly designed setups were clinically verified during discussions with surgeons and the nursing staff. In sections OR setups for total knee replacement and OR setups for total hip replacement, the initial and newly developed setups for THA and TKA surgeries are presented and discussed in detail.

### Simulation experiments

**Fig. 3:**
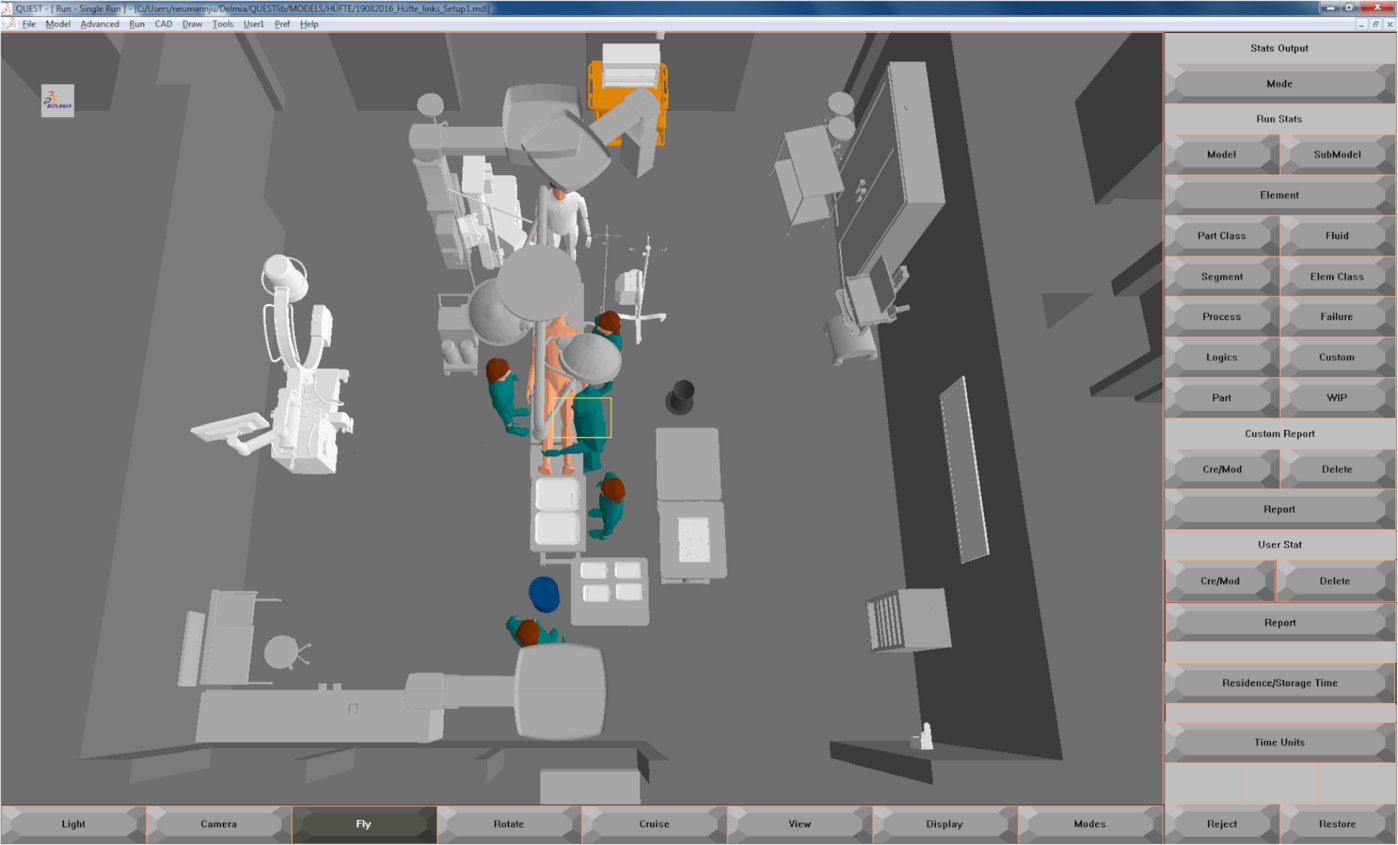
Example of a 3D simulation model of left-side THA Setup 1 (created with Delmia Quest, Dassault Systèmes, version V5-6R)

For every recorded and newly designed OR setup, a simulation model was created with Delmia. Figure 3 shows an example of the Delmia simulation environment with a 3D model of the orthopedic OR for a left-side THA setup. Due to the different setups according to the operated leg of the patient, in each case, a left-side and right-side setup were modeled. This results in 6 THA simulation models (2 initial setups and 1 newly designed setup) and 8 TKA simulation models (1 initial setup and 3 newly designed setups). The TKA/THA setups are described in detail in sections OR setups for total knee replacement and OR setups for total hip replacement. For all models, a simulation study was performed in which the instrument handover between the surgeon and the scrub nurse as well as the travel path of the circulator were analyzed. Thereby, the initial setups could be objectively compared to new setups without the need to implement the setup alternatives directly in the intraoperative environment. A detailed description of the conducted simulation experiments can be found in Appendix B.

### Analysis

In the next step, a statistical analysis was performed based on the simulation results. Thereby, the initially recorded setups, which were used at the University of Leipzig Medical Center, were compared with the newly developed setups to determine an improved intra-OR layout for THA and TKA surgeries. For every setup and instrument table the instrument handover times, and the total instrument handover times of a surgical intervention were simulated and compared. Additionally, the TRM was calculated and the travel paths of the circulator were simulated in Delmia for right-side and left-side setups. The results were analyzed in the in-silico-comparison, which is presented in section In silico comparison of initial and newly designed OR setups. The THA and TKA setups, which performed best in the simulation scenario were validated and discussed with the surgical team and a final adaptation was made.

### Evaluation

The THA and TKA setup, which performed best in the simulation scenario was implemented in the orthopedic OR. Thereby, in 2018 15 THA and 14 TKA surgeries were recorded at the Division of Endoprothetic Joint Surgery and General Orthopaedics at the University of Leipzig Medical Center. The number and duration of instrument handover, the incision-to-begin-of-closure time (IBCT) as well as the incision-to-closure-time were recorded for every surgery. In further data analysis and setup evaluation (Evaluation of the best-performing setup in the actual intraoperative OR environment), only the surgery duration until the end of the last interventional activity and the beginning of the suturing is considered. The suturing is often performed by a novice, which results in a higher amount of instrument handovers and longer durations for suturing. Therefore, only the IBCT is considered to ensure better comparability. Subsequently, the data were compared with the simulation results and evaluated against the data, recorded in the initial THA and TKA setups (Inter-setup evaluation of the simulation results).

## Results

### OR setups for Total knee replacement

#### Initial TKA setup 1

**Fig. 4:**
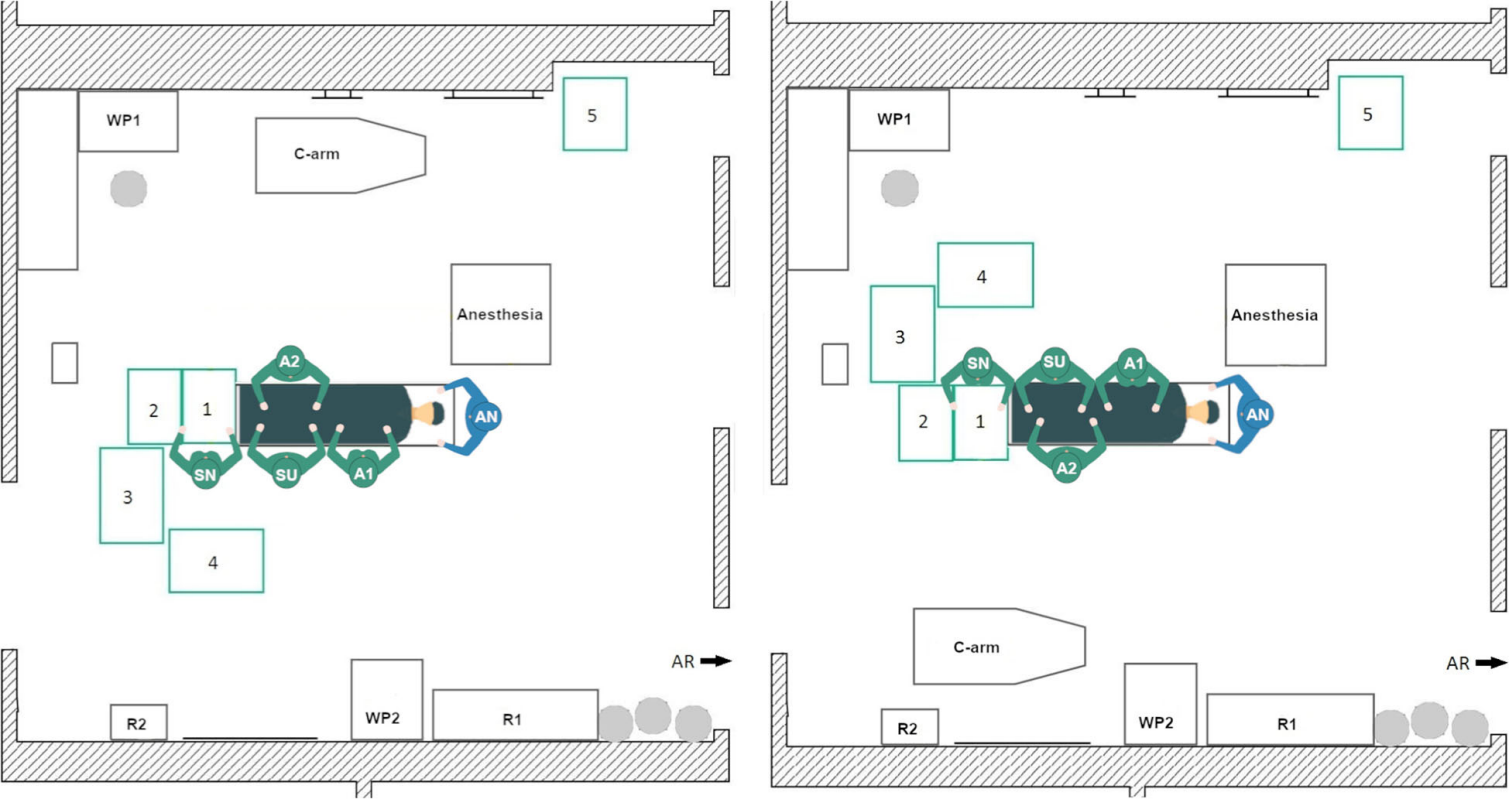
Schematic representation of the left side TKA Setup 1 (left) and right-side TKA Setup 1 (right), (SN – scrub nurse; SU – surgeon; A1, A2 – 1st and 2nd assistant; AN – anesthesiologist; R1, R2 – rack with materials and supplies; WP1, WP2 – working place with displays; AR – anesthesia room)

During data acquisition, the initial TKA Setup 1 was used with minor variations in the position of the 1st assistant in every TKA surgery at the orthopedic department. In the left-side TKA Setup 1 (Fig. 4, left) the scrub nurse (SN), surgeon (SU) and the 1st assistant (A1) stand on the left side of the OR table as seen by the patient. The A1 stands either on the same site as the surgeon or together with the 2nd assistant (A2) on the opposite. The instrument Tables 1–4 are located at the lower end of the OR table in a U-shape around the scrub nurse. In some surgeries, Table 5 is used as a backup table. The c-arm is used during surgery for the evaluation of the implant position. It is located behind the A2 and is brought to the right side of the OR table when needed. The setup is mirrored along the operating table for the right-side TKA (Fig. 4, right).

A beneficial factor is that the scrub nurse has a direct view of the operating area and has a good position for the instrument handover with the surgeon. Disadvantages arise from the need of the scrub nurse to turn around to reach Tables 2, 3 and 4. Thereby, the direct view to the surgeon and the operating area is blocked. In the right-side setup, the instrument tables are close to the working place (WP1), which indicates that the circulator is often passing the instrument tables nearby. This entails air movement and a potential threat to the sterility of the instruments and implants. For the left-side setup, there is also little space between the WP1 and Tables 2 and 3, when the circulator is going to the sterile room on the left side of the OR.

#### Newly designed TKA setup 2

**Fig. 5:**
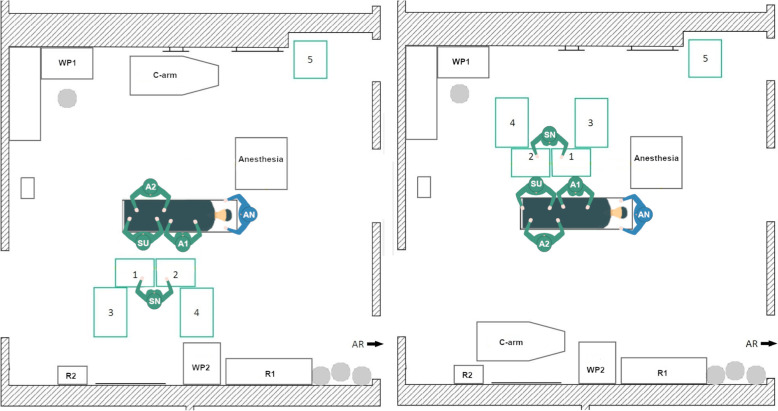
Schematic representation of the left-side TKA Setup 2 (left) and the right-side TKA Setup 2

In this newly designed left-side TKA setup (Fig. 5, left) the rotational movement of the scrub nurse should be reduced by arranging the instrument tables in a U-shape behind the surgeon. Thereby, Tables 1 and 2 are located between the scrub nurse and the surgeon. Tables 3 and 4 are located on the left and the right side of the scrub nurse.

In the right-side setup (Fig. 5, right) there is little space for the circulator between the WP1 and the instrument tables. In the left-side setup, workplace 2 is almost completely blocked by an instrument table. In addition, the travel path is entirely impassable to one side, but all destinations can be reached. Another disadvantage is that the scrub nurse stands behind the surgeon, which leads to an increase in the rotational movements of the surgeon and possibly has a negative impact on the hygiene. Nevertheless, the setup was considered for further evaluations.

#### Newly designed TKA setup 3

**Fig. 6:**
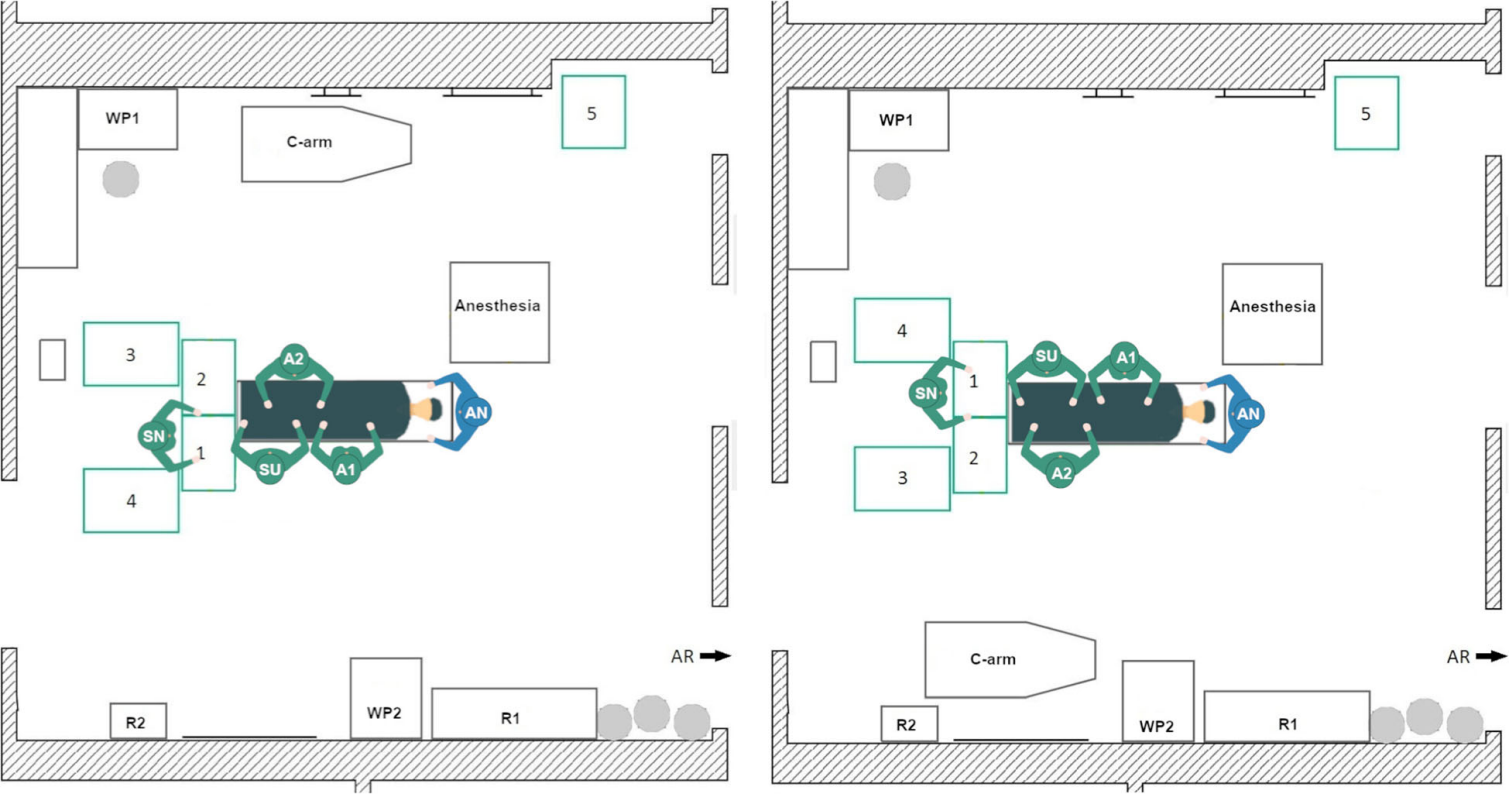
Schematic representation of the left-side TKA Setup 3 (left) and right-side TKA Setup 3 (right)

The goal of the TKA Setup 3 design was to maintain the compact table layout of Setup 2 but to establish a direct view between the surgeon and the scrub nurse as well as the operating area. Therefore, in the left-side TKA Setup 3 (Fig. 6, left) the instrument tables are located in a U-shape around the scrub nurse at the bottom of the OR table. The scrub nurse faces the surgeon directly and has a direct view of the operating area. The circulator can reach all workplaces and supply storage without any obstructions. The right-side THA Setup 3 (Fig. 6, right) is mirrored along the operating table.

#### Newly designed TKA setup 4

**Fig. 7:**
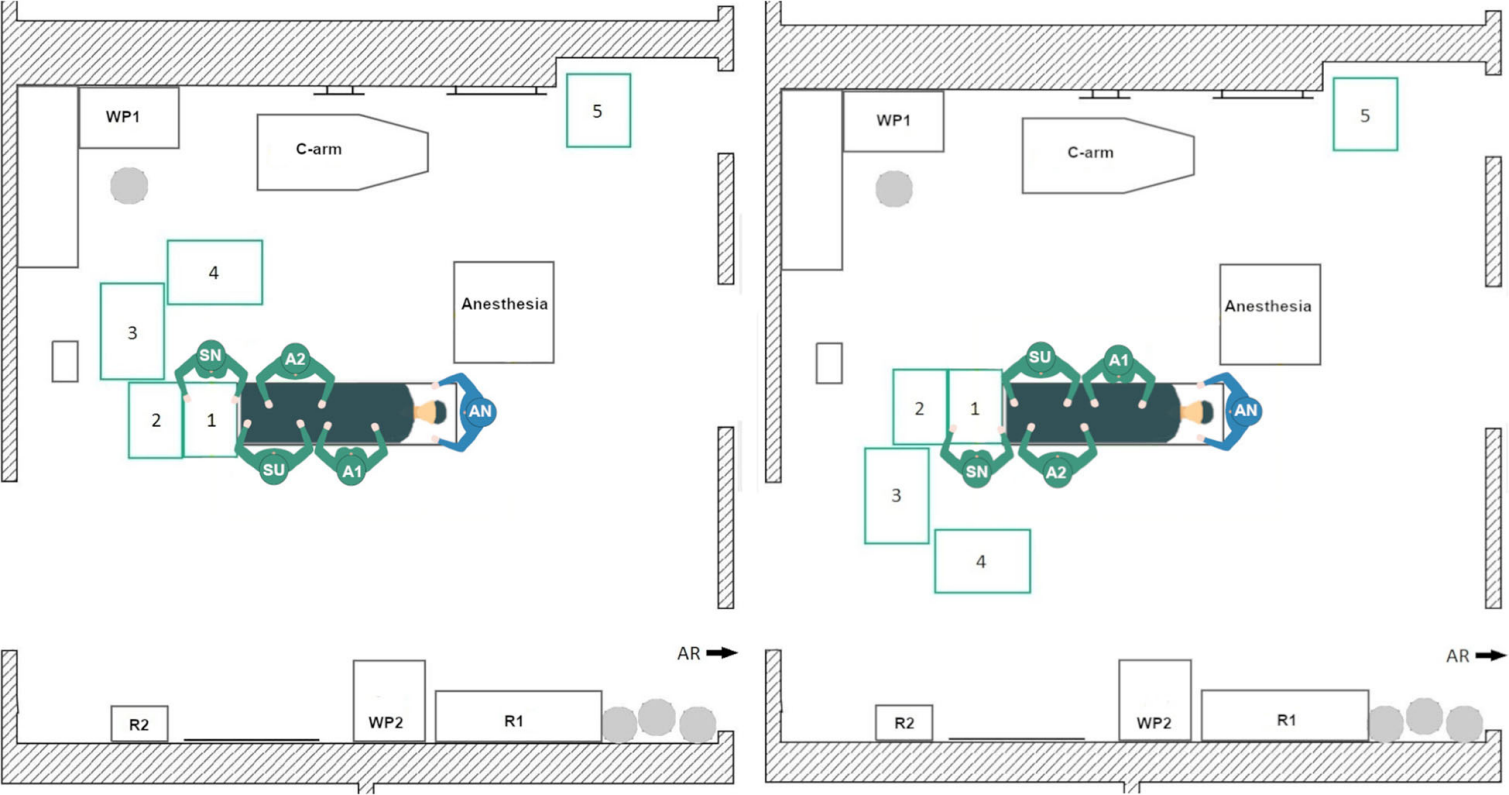
Schematic representation of the left-side TKA Setup 4 (left) and right-side TKA Setup 4

The newly designed TKA Setup 4 is inspired by the table layout of the initial TKA Setup 1, but the setup differs in the position of the scrub nurse. In the new TKA Setup 4 (Fig. 7) the scrub nurse is facing the surgeon by standing on the opposite side of the OR table, instead of next to the surgeon. Thus, a direct view can be established between the two persons and the operating area is also directly visible to the scrub nurse. This does not solve the problem that the scrub nurses’ view is blocked when turning to Tables 3 and 4. In addition, the tables are close to the WP1 of the circulator.

### OR setups for Total hip replacement

#### Initial THA setup 1

**Fig. 8:**
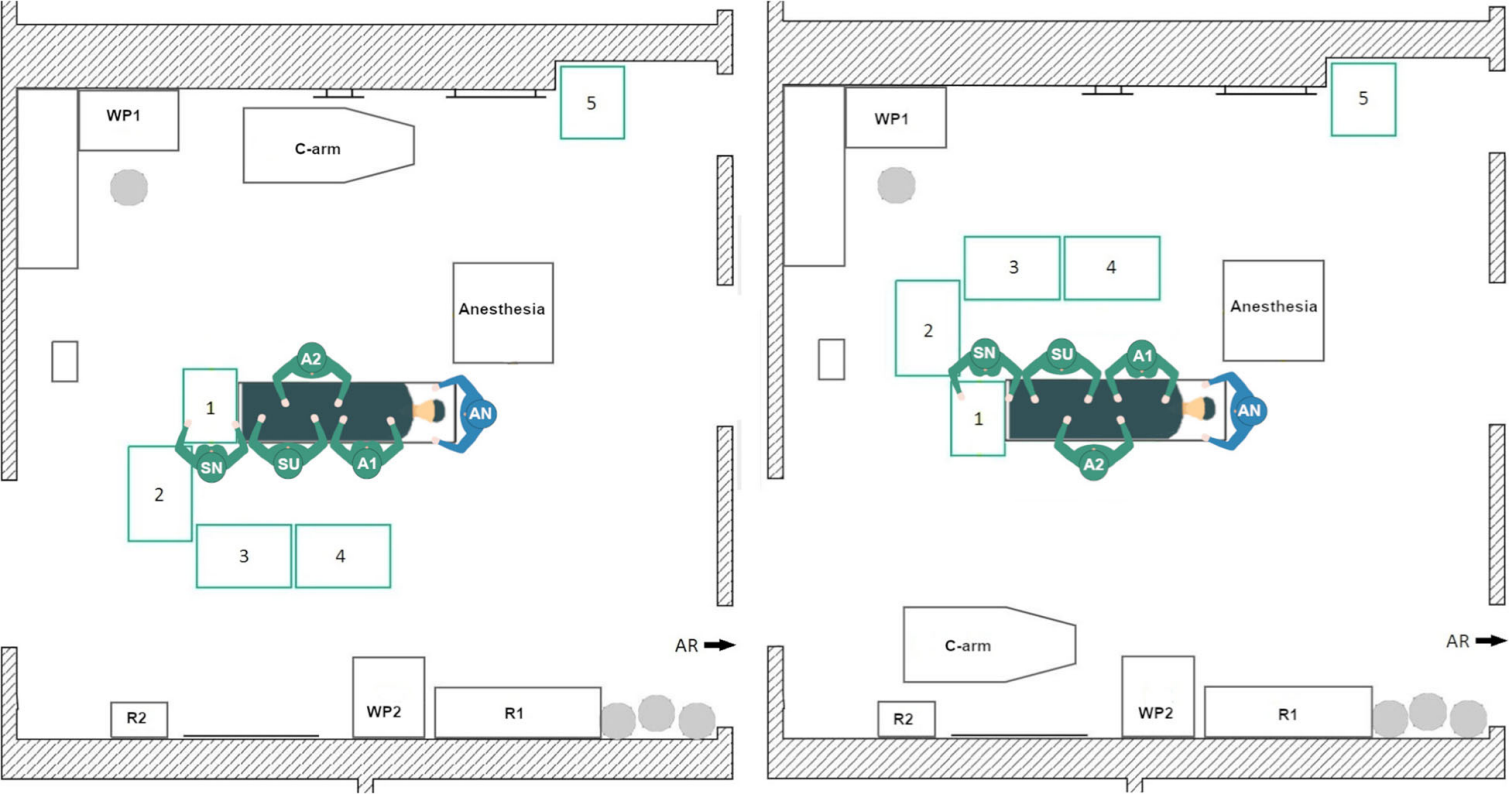
Schematic representation of the left-side THA Setup 1 (left) and right-side THA Setup 1 (right)

For left-side THA (Fig. 8, left), the scrub nurse, the surgeon, and the 1st assistant are positioned on the left side of the patient’s body, while the 2nd assistant stands on the right side (healthy hip joint). The surgeon stands at the patient’s hip. The four tables are arranged in a J-shape around the scrub nurse at foot of the OR table with one table in front, on the left side and two tables behind. The right-side THA Setup 1 is mirrored along the operating table (Fig. 8, right).

The main advantage is that the scrub nurse is in a good position to the surgeon for instrument hand over. Thus, the direct view of the operating area is occasionally limited when the surgeon turns to the patient or the scrub nurse turns to Tables 2, 3 and 4. For the right-side THA setup, the instrument tables are located with less than 1 m distance to the WP1, which can affect the sterility of the instrument tables. For the left-side THA setup, there is also little space between WP2 and Table 4, which impedes the direct travel path of the circulator to the supply storage (R1 and R2) as well as the working place 2 (WP2).

#### Initial THA setup 2

**Fig. 9:**
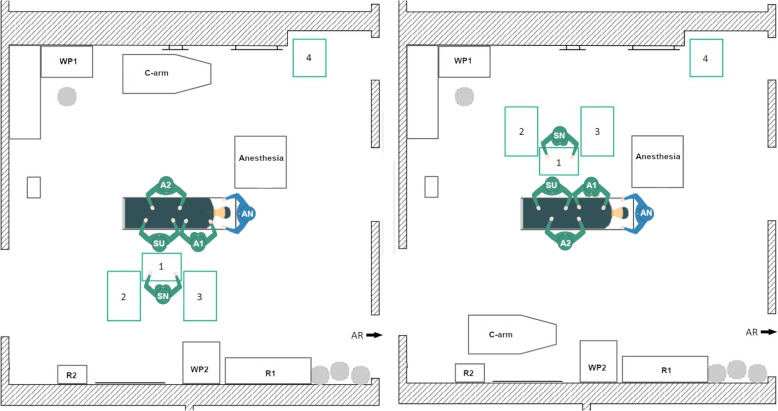
Schematic representation of the left-side THA Setup 2 (left) and the right-side THA Setup 2

In the second recorded THA setup, there are only three instrument tables, which are arranged in a U-shape around the scrub nurse behind the surgeon with Table 1 between them. The use of a 3-table or 4-table setup depends on the preferences of the OR team as well as resource requirements and the complexity of the surgery. In principle, a 3-table setup is sufficient for most of the THA surgeries. Therefore, an additional table is positioned on the left side and one on the right side of the scrub nurse. For left-side THA (Fig. 9, left), the surgeon, scrub nurse, and the 1st assistant are located on the left side of the patient and the 2nd assistant stands on the right side. For right-side THA the OR setup is mirrored along the OR table (Fig. 9, right).

In the initial THA Setup 2, half of the OR is blocked for the circulator, but the travel paths to the anesthesia room on the right side and the sterile room on the left side of the OR can be reached without close proximity to the operating area and the instrument tables. Another advantage is that the scrub nurse has a direct view of the operating area and the surgeon even when turning to Tables 2 and 3. The slight drawback of the setup is that the surgeon has to turn around for every instrument handover.

#### Newly designed THA setup 3

**Fig. 10:**
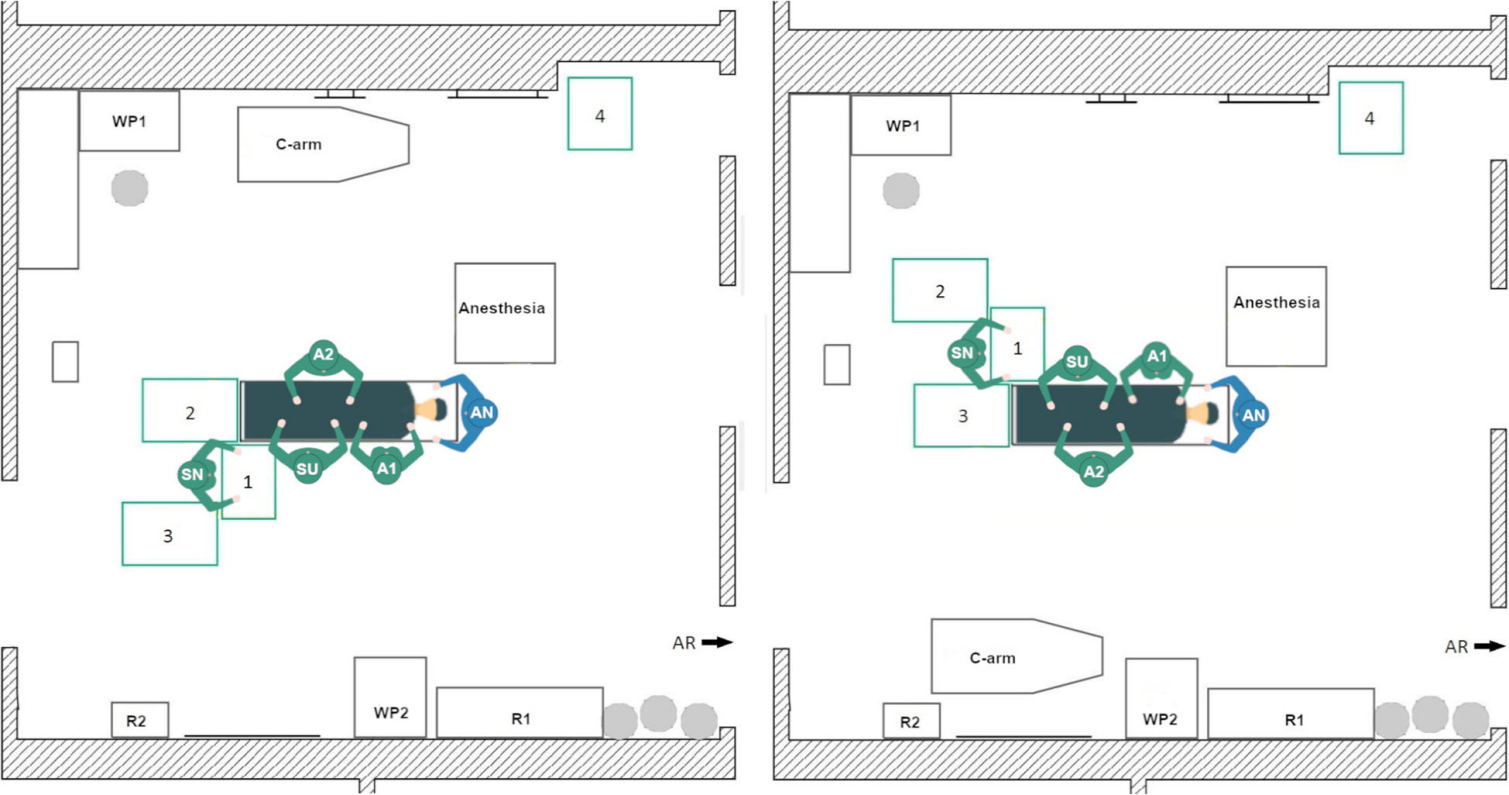
Schematic representation of the left-side THA Setup 3 (left) and right-side THA Setup 3 (right)

For THA surgery one OR setup is newly designed with only three instrument tables. In the left-side THA Setup 3 (Fig. 10, left), the instrument tables are located around the scrub nurse. Table 2 is positioned directly at the bottom of the OR table. Table 1 stands between the scrub nurse and the surgeon. Additionally, Table 3 closes the U-shaped arrangement around the scrub nurse, who is facing the surgeon directly. The circulator has unrestricted access to all supply stocks and workplaces. For right-side THA (Fig. 10, right), the setup is mirrored along the operating table.

### In Silico comparison of initial and newly designed OR setups

#### Instrument handover times

**Fig. 11:**
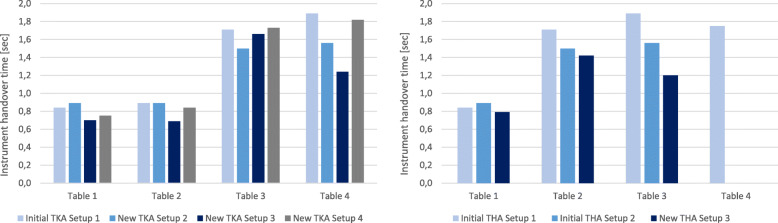
Comparison of Delmia simulation results for the instrument handover times of the TKA Setups 1-4 and tables 1-4 (left side) and THA Setups 1-3 and tables 1-4 (right side)

The instrument handovers for all tables in all TKA setups were simulated with Delmia and subsequently compared with each other (Fig. 11, left side). As a result, TKA Setup 3 enables the fastest instrument handover between scrub nurse and surgeon for instrument Tables 1, 2 and 4. For instrument Table 3 TKA Setup 2 allows a slightly faster handover. TKA Setup 1 and Setup 4 have equivalent instrument handover times due to the similar setup design whereby TKA Setup 4 is slightly faster for the instrument Tables 1, 2 and 4.

For all tables and THA setups, the instrument handover times were also simulated with Delmia and then compared with each other (Fig. 11, right). The newly designed THA Setup 3 enables the fastest instrument handover between the surgeon and the scrub nurse for all tables.

#### Total instrument handover times

**Fig. 12:**
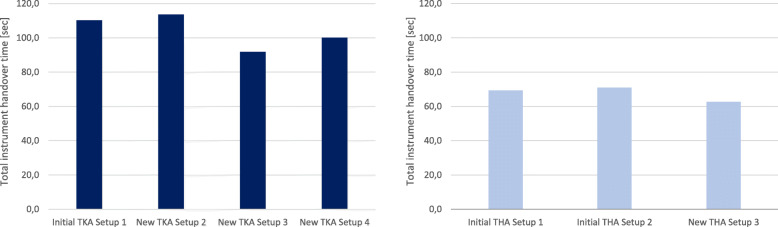
Comparison of Delmia simulation results of the total instrument handover time for the TKA Setups 1-4 (left side) and THA Setups 1-3 (right side)

During initial data acquisition, the total number of instrument handovers and the handover path in 7 TKA surgeries, were recorded in the initial TKA Setup 1. For instrument Table 1 an average of 108.3 ± 32.2 instrument handovers, for instrument Table 2 an average of 5.7 ± 7.3 handovers, for instrument Table 3 an average of 5.0 ± 4.2 handovers and for the instrument Table 4 an average of 2.7 ± 2.3 instrument handovers were documented. Based on the number of handovers, the total handover time was calculated in the Delmia simulation scenarios for TKA setups (Fig. 12, left). As a result, TKA Setup 3 performed best with a total instrument handover time of 91.8 s. TKA Setup 2 was the slowest setup in the simulation. There was no measurable difference between right-side and left-side setup in the simulation of total instrument handover times.

For the THA interventions, the total number of instrument handovers and the handover path were recorded. Thereby, 9 THA surgeries with Setup 1 and 6 THA surgeries with Setup 2 were recorded. In THA Setup 1, an average of 60,8 ± 19.1 instrument handovers was recorded from instrument Tables 1, 2.2 ± 2.5 handovers from instrument Tables 2, 1.7 ± 1.5 handovers from instrument Table 3 and 1.4 ± 2.5 handovers from instrument Table 4. In THA Setup 2, an average of 71.7 ± 22.6 instrument handovers was recorded from instrument Tables 1, 9.2 ± 8.5 from instrument Tables 2 and 0.3 ± 0.8 handovers from instrument Table 3. Based on the average handovers from THA Setup 1 and Setup 2, the total handover time was calculated with Delmia for THA setups (Fig. 12, right). In the simulation scenario, THA Setup 3 performed best with a total instrument handover time of 62.6 s.

#### Distance traveled by the circulator

During the initial data acquisition, the number and pathways of the circulator were recorded in the 7 TKA surgeries. Thereby, the circulator travels on average 4.0 ± 1.6 times to the supply rack R1, 2.7 ± 2.0 times to supply rack R2, 2.7 ± 1.4 times to the supply stock ST1, which is located outside of the OR, 3.0 ± 0.8 times to the supply stock ST2, which is located in the sterile room on the left side of the OR and 2.2 ± 1.2 times to the anesthesia room on the right side of the OR. Additionally, the circulator walks on average 7.6 ± 3.0 times to the scrub nurse to hand over sterile supplies or for the support of material opening.

**Fig. 13:**
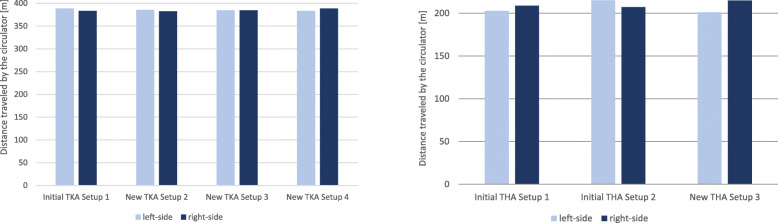
Total distance traveled by the circulator during one surgery with TKA Setup 1-4 (left side) and THA Setup 1-3 (right side)

The simulation results of the traveled distance (Fig. 13, left) showed that there are only slight differences between the setups. The walking distance is marginally longer, mainly for the left-side TKA, if the instrument tables are blocking the pathway between the WP1 and the scrub nurse as well as the supply racks R1 and R2. Setup 3 provides the best balance of left-side and right-side setup. Thus, the travel paths do not differ for left-side and right-side TKA, since the position of the tables does not change as a result of the mirroring.

On the right side of Fig. 13, the total distance traveled by the circulator during a THA surgery is presented. The number of pathways was recorded in 15 THA surgeries. The circulator travels on average 2.6 ± 2.9 times to the supply rack R1, 1.5 ± 1.1 times to the supply rack R2, 0.3 ± 6.6 times to the supply stock ST1, 4.5 ± 2.3 times to the supply stock ST2 and 2.3 ± 1.6 times to the anesthesia room as well as 5.0 ± 4.7 times to the scrub nurse. As a result of the Delmia simulation scenario, the walking distance of the left-side THA Setup 3 is the shortest. For the right-side Setup 3, it is marginally longer than Setup 1 and the right-side Setup 2. For the right-side THA Setup 3, the circulator needs to go around the operating room table due to a blocked passage on the left side of the OR, which leads to a slightly longer pathway compared to the initial THA Setups 1 and 2.

#### Total rotational movement

**Fig. 14:**
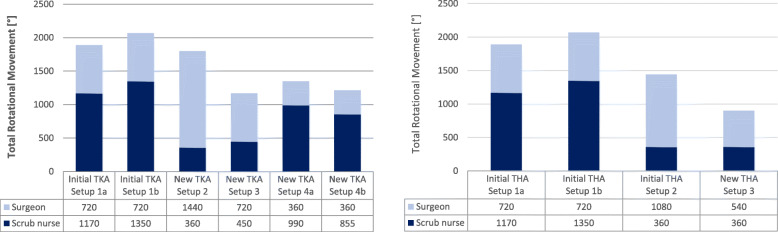
Total Rotational Movement for the TKA Setups 1a-4b and THA Setups 1a-3. Setups marked with a are calculated with a rotation to the right side and Setups marked with b are calculated with a rotation to the left-side

In the left-side Fig. 14, the TRM for all TKA setups is presented. For the initially recorded TKA Setup 1, the theoretically calculated TRM is 1890° (Setup 1a - rotation to the right side) and 2070° (Setup 1b - rotation to the left side), for the newly designed TKA Setup 2 the TRM is 1800° and for TKA Setup 4 the TRM is 1350° (Setup 4a -rotation to the right side) and 1215° (Setup 4b - rotation to the left side). TKA Setup 3 has the lowest TRM with 1170° and seems to be the most balanced solution between the TRM of the surgeon and the scrub nurse.

The TRM of all THA setups is presented in the right-side Fig. 14. For the initially recorded THA Setup 1, the TRM is 1890° (Setup 1a - rotation the right side) and 2070° (Setup 1b - rotation to the left side) and for THA Setup 2 the TRM is 1140° for every instrument hand over. The newly designed THA Setup 3 has the lowest TRM with 900° and provides a good balance between the TRM of the surgeon and the scrub nurse.

### Evaluation of the best-performing setup in the actual intraoperative OR environment

**Fig. 15:**
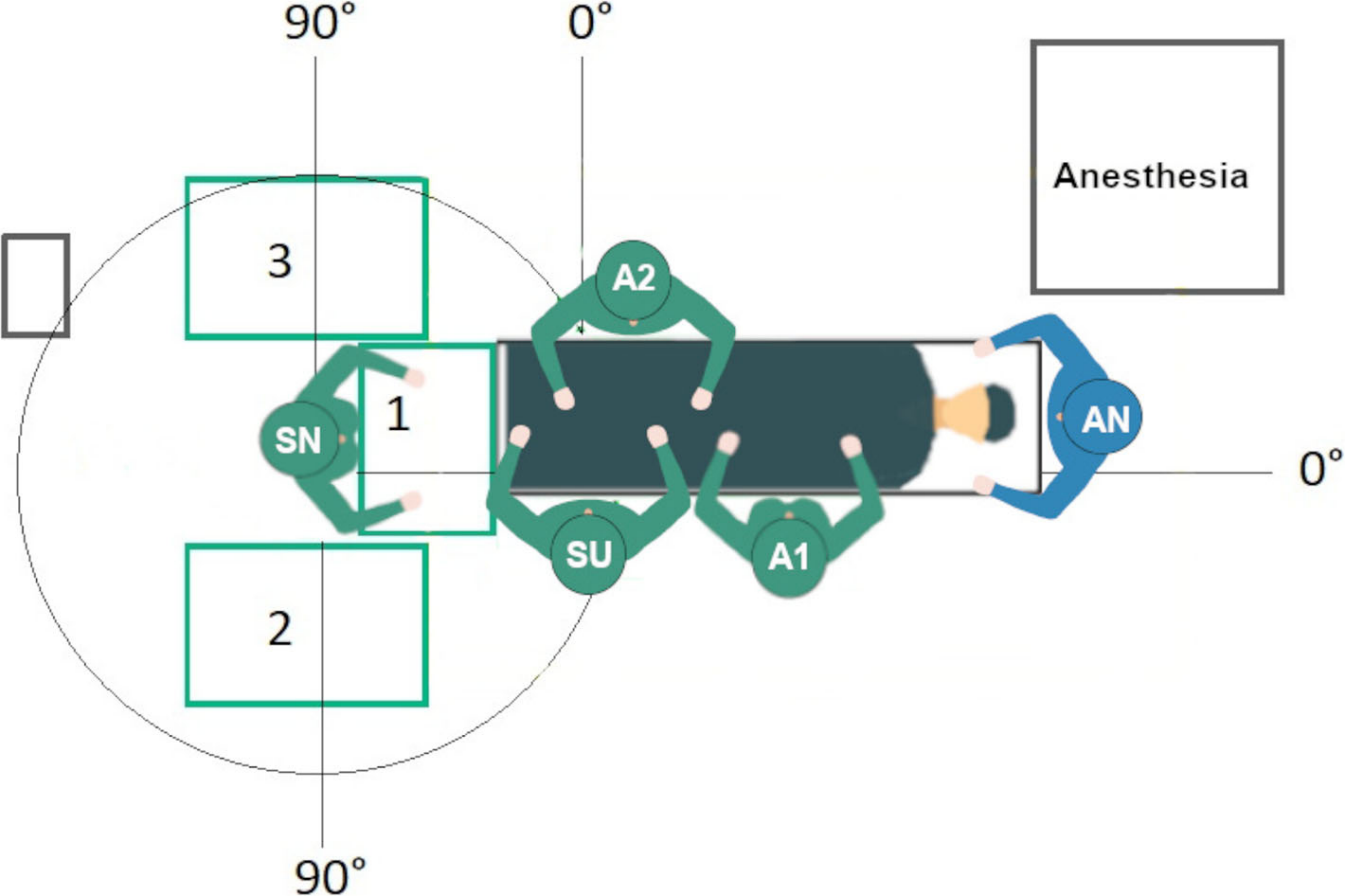
Schematic representation of the final TKA Setup 3, which was evaluated in the actual OR setting

For TKA surgery, Setup 3 seemed to be the best option of all analyzed TKA setups. Instrument handover times, TRM, as well as the travel paths of the circulator, performed better than the other designed setups and the initial Setup 1. The tables are arranged in a U-shape around the scrub nurse at the bottom of the OR table. Beneficially, the scrub nurse faces the surgeon directly and has a direct view on the operating area. Furthermore, the circulator can reach all workplaces and supply storage without any obstructions. The instrument table setup was discussed with the surgical team of the Division of Joint Replacement and Orthopedics and a final adaptation was made before the setup was implemented in the actual OR and daily clinical routine. This led to a slightly adapted OR layout with only 3 instrument tables for TKA surgery (Fig. 15).

For THA surgery, also Setup 3 seems to be the best option of the analyzed setups. The single and the total instrument handover times, TRM, as well as travel paths of the circulator, showed better results than both initial setups. In Setup 3 the three instrument tables are arranged in U-shape around the scrub nurse at the bottom of the OR table. The scrub nurse faces the surgeon and has a direct view of the operating area. Additionally, the circulator has unrestricted access to all destinations.

**Fig. 16:**
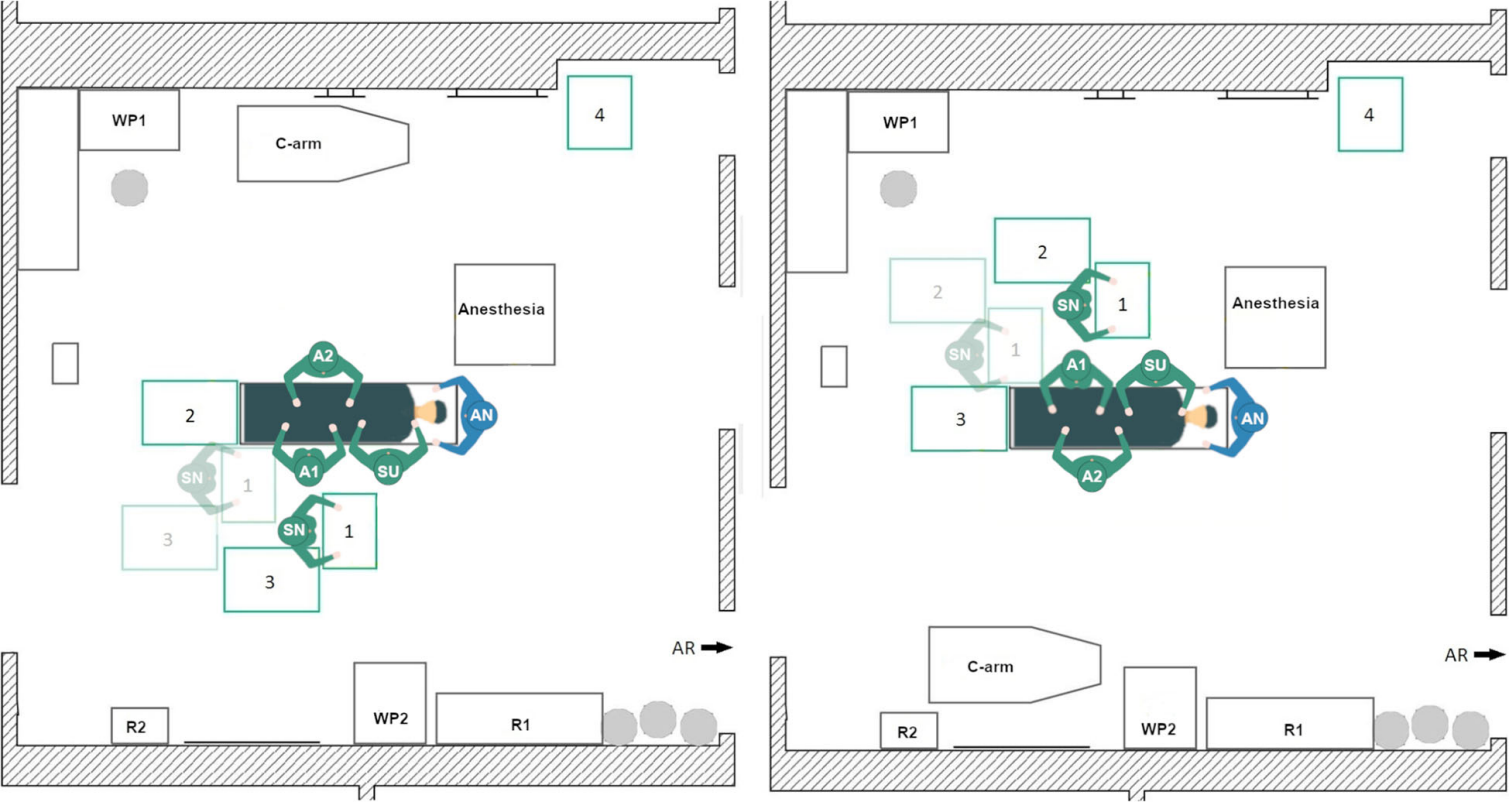
Schematic representation of the final left and right-side THA Setup 3 during position change of the surgeon and 1st assistant

THA Setup 3 was also discussed with the orthopedic OR team before it was implemented in the intraoperative OR environment. Thereby, a working method for the position change of the surgeon and the 1st assistant was identified (Fig. 16). The challenge was the rearrangement of the instrument tables during the insertion of the femur part of the hip implant. The scrub nurse should be able to reach the surgeon within an arm length, although the 1st assistant stands between them. For this purpose, the scrub nurse pushes the instrument Tables 1 and 3 (left-side THA) respectively Tables 1 and 2 (right-side THA) forward between the 1st assistant and the surgeon. Thereby, all instruments needed for the insertion of the femur implant need to be available at the two moving tables. After the femur implant is inserted, the surgeon, 1st assistant and scrub nurse change back to the initial position.

The setups, which performed best in the simulation scenarios, were evaluated in the actual intraoperative OR environment and additionally 14 TKA and 15 THA were recorded. Therefore, the time of instrument handovers, as well as the IBCT, were documented and compared to the initial intraoperative data.

#### Instrument handover times

**Fig. 17:**
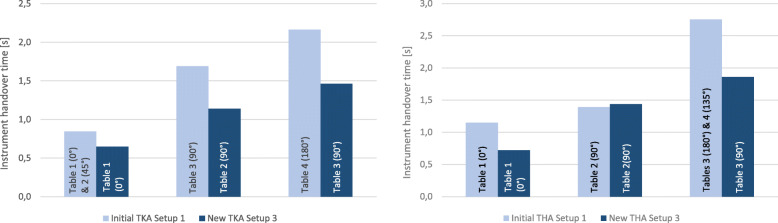
Comparison of the intraoperative instrument handover times of TKA Setup 1 & 3 (left side) and THA Setup 1 & 3 (right side)

Firstly, the instrument handover times of the new TKA setup were compared to the initial measured data of the TKA Setup 1 (Fig. 17, left). The instrument Tables 1 and 2 were combined in the final TKA Setup 3 (Fig. 15) to one 0°-table. Consequently, the measured handover times of the initial TKA Setup 1 for instrument Table 1 (0°) and 2 (45°) were merged for better comparability with the new three-table TKA setup. The 90° table in TKA Setup 1 was compared with the left-side 90° Table 2 in Setup 3. Since the 180° instrument table in Setup 1 and the right-side 90° table in Setup 3 involve the same instruments, both tables were compared with each other. The instrument handovers in Setup 3 are faster for all 3 instrument tables. Although, statistical significance has not been reached for instrument Table 2 (*p* = 0.121) and Table 3 (*p* = 0.102), a statistical significance in a two-tailed t-test have been reached for instrument Table 1 (*p* = 0.004).

The instrument handover times of the new THA Setup 3 were compared to the initial data measured in the initial THA Setup 1 (Fig. 17, right). Table 3 and Table 4 of THA Setup 1 were combined for better comparability with the three-table THA Setup 3. The measured handover times of the initial THA Setup 1 for Table 1 were compared with Table 1 of THA Setup 3. Table 2 of THA Setup 1 was compared with the left-side Table 2 of THA Setup 3. Due to the equivalent instrument usage, Table 3 and Table 4 of THA Setup 1 were compared with the right-side Table 3 of THA Setup 3. The instrument handovers in THA Setup 3 are faster for Tables 1 and 3. A statistical significance of Setup 3 in a two-tailed t-test could be shown for Table 1 (*p* = 0.0001) and Table 3 (*p* = 0.047).

#### Incision-to-begin-of-closure time

**Fig. 18:**
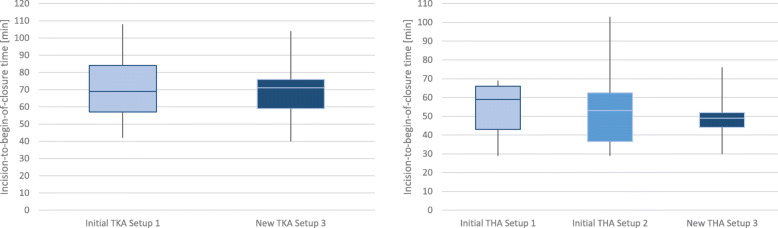
Comparison of IBCT for TKA Setup 1 and 3 (left side) and THA Setup 1, 2 and 3 (right side)

Secondly, the impact of the improved instrument handover time has an impact on the overall efficiency of the surgical procedure was analyzed. Therefore, the IBCT of TKA Setup 3 was recorded and compared to the initial IBCT performance of the TKA Setup 1 (Fig. 18, left). In surgeries performed with TKA Setup 1, the mean IBCT is 71.1 ± 20.7 min. In surgeries performed with the new TKA Setup 3, the mean IBCT was slightly faster with 70.7 ± 17.1 min. Although the variance of procedures performed with TKA Setup 3 is lower and indicates a faster and more efficient surgery, there is no significant difference in the duration of the procedure for TKA surgeries (*p* = 0.94).

The IBCT was also measured for both initial THA setups and subsequently compared to the intraoperatively recorded IBCT of THA Setup 3 (Fig. 18, right). The mean IBCT is 53.2 ± 14.7 min for surgeries performed with THA Setup 1, 54.5 ± 22.8 min for THA Setup 2 and 49.7 ± 12.2 min for THA Setup 3. Although the newly designed setup performs on average 3.49 min faster than THA Setup 1 and 4.77 min faster than THA Setup 2, a statistical significance was not reached (Setup 1 *p* = 0.53, Setup 2 *p* = 0.50).

### Inter-setup evaluation of the simulation results

In the last step, the Delmia simulation results of the newly designed THA Setup 3 and TKA Setup 3 were compared with the intraoperatively recorded data to evaluate the accuracy and closeness to the actual intraoperative situation. For this purpose, the instrument handover time was analyzed.

The simulated and real intraoperative instrument handover times (IHT) of TKA Setup 3 and THA Setup 3 are presented in Table 2. The difference between the simulation and real OR IHT is exceptionally small for all tables in TKA Setup 3 and Tables 1 and 2 in THA Setup 3. Only Table 3 in THA Setup has a higher deviation between simulation and the real OR measurement of 0.66 s. Nevertheless, the results indicate high accuracy and closeness of the Delmia simulation to the actual intraoperative situation. This enables a valid evaluation of setup changes by a computer simulation before their implementation in the actual OR environment.

**Table 2:** Comparison of Delmia simulation results and intraoperative measurements of instrument handover times

	***TKA Setup 3***			***THA Setup 3***		
	IHT Delmia simulation [sec]	IHT intraoperative measurement [sec]	Δ [sec]	IHT Delmia simulation [sec]	IHT intraoperative measurement [sec]	Δ [sec]
Table 1	0.70	0.65	0,05	0.79	0.72	0.07
Table 2	1.24	1.14	0,10	1.42	1.44	0.02
Table 3	1.66	1.46	0,20	1.20	1.86	0.66

## Discussion

The improved TKA Setup 3 and THA Setup 3 were permanently implemented in the daily clinical routine at the University of Leipzig Medical Center. In particular, the surgeons and scrub nurses acknowledged the newly designed setups’ good operability and improved ergonomics. In a questionnaire study, the OR personnel indicates a high level of satisfaction with the improved setups [63]. For the TKA setup, the scrub nurses especially like the good view of the operating area and the surgeon, which enables a faster and proactive instrument handover. The surgeons have improved accessibility to the instrument tables and often perform instrument handover without any rotations, which consequently improves the ergonomics of the setup. The same applies to the improved THA setup. The surgeon does not need to turn or bend the upper body, to grab an instrument. Also, the scrub nurses describe the setup after the position change of the surgeon and the 1st assistant as good manageable.

One of the main benefits of the presented methodology is the strong user involvement in the development process and the simulation study. With the help of the 3D graphical representation, the clinical stakeholders were able to contribute their expertise in the design of the new OR setups, which enables the intraoperative implementation of efficient OR layouts with high user acceptance. The new setups were also analyzed with respect to hygienic considerations and were assessed positively by the Institute of Infection Control and Hospital Hygiene of the University Medical Center Leipzig.

Besides the positive subjective assessment of the improved setups, an objective assessment has been performed in this study. It was shown, that the newly designed setups perform better in the simulation environment as well as in the intraoperative setting. In the simulation, the instrument handover times, total instrument handover times and travel path of the circulator could be slightly reduced. In the intraoperative setting, the instrument handover time, surgery duration, and the ergonomic situation, team collaboration, and intraoperative hygiene could be improved by the implementation of the enhanced OR setups. Additionally, the simulation results strongly correlate to the intraoperative measured data. Therefore, the developed methodology for the design, simulation, analysis, and evaluation of improved OR setups has proven its suitability for the intended application. The methodology has been successfully applied to different orthopedic intervention types (THA and TKA) and could be adapted to other interventions and surgical disciplines. With the simulation-based methodology, it is possible to analyze different possible setups from different perspectives. This allows a valid assessment of the performance prior to the implementation of untested setups in the intraoperative setting, which could lead to adverse effects. Intraoperative processes are highly variable, complex, intertwined and have a significant impact on each other. Therefore, a change in the intraoperative setting should not be tested ad-hoc without prior considerations and tests. The proposed methodology enables the design and assessment of improved OR setups for different surgical disciplines tailored to the procedural, structural and personnel characteristics of a specific OR and clinic. However, the introduced THA and TKA setups are basically applicable to other similar equipped ORs, if the structural, and procedural requirements are met.

A slight drawback of the presented methodology is the high effort for data acquisition and simulation implementation. Nevertheless, the DES enables an objective performance evaluation of the analyzed setups from different perspectives, which would not have been possible solely on the base of the intraoperative measurements. Additionally, the 3D representation provides an adequate environment for the design of the new setup alternatives considering the requirements for the improvement of THA and TKA setups. Also, the animated 3D models provide the base for the involvement of different clinical stakeholders in the group-based decision-making process. Overall, the study shows that the results are worth the effort due to the improvement of the surgeries’ efficiency. The new THA setup is about 4 min faster than the initial setups. In [64] it was shown, that also small improvements in IBCT in combination with a perioperative business process reengineering could to lead a better balancing and improved utilization of available OR capacities.

## Conclusion

Modern operating rooms are the most cost-intensive but also a high-income department in the hospital. The surgical department is in the focus of continuous optimization to create an efficient and safe environment for optimal patient treatment. Due to the complexity and sensitivity of perioperative processes, changes in the intraoperative setting should not be tested ad-hoc without prior considerations and tests. Simulation models provide an effective approach for the improvement of processes and structures. Additionally, the simulation could indicate in which way processes need to be adapted and how the process efficiency is changed due to the impact of different procedural, behavioral, structural, operational or temporal parameters.

In this work, a methodology was presented for the design, analysis, simulation, and evaluation to determine improved OR setups tailored to a specific surgical intervention. For the use case of TKA and THA surgeries, enhanced OR setups were designed, and analyzed form different perspectives based on discrete event simulation. The simulation results were evaluated in the actual intraoperative OR setting. Thereby, the instrument handover time could be reduced for all instrument tables in the newly designed TKA setup and statistical significance was shown for the primarily used Table 1. For the improved THA setup, the instrument handover times are significantly faster for the main instrument Table 1 and Table 3. Comparing the setups in the simulation scenario, the travel paths of the circulator could be slightly reduced, and the ergonomic situation of the OR team was improved. With the implementation of the improved THA and TKA setups in the actual OR environment, the incision-to-begin-of-closure-time could be reduced. An inter-setup evaluation was performed to demonstrate the accuracy of the method. The results indicate high accuracy and closeness of the simulation model to the actual intraoperative situation. This enables a valid evaluation of setup changes before their implementation in the actual OR environment. The strong user involvement in the development process results in the implementation of efficient OR setups with high user acceptance. Therefore, the presented method proved to be suitable for the presented use case but provides also high flexibility for other optimization objectives concerning the OR layout. Additionally, the method can also be adapted to further intervention types and surgical disciplines.

## Supplementary information

**Additional file 1 Supplementary file 1.** Appendix A – Simulation Implementation. The supplementary data consists of a detailed description of the used software simulation tool (Delmia Quest by Dassault Systèmes) and the implementation of the simulation environment.

**Additional file 2 Supplementary file 2**. Appendix B – Simulation Experiments. The supplementary data presents the conducted simulation experiments in detail. Especially, the simulation of the instrument handover times between scrub nurse and surgeon as well as the simulation of the circulators’ travel path is described.

## Data Availability

The datasets used and/or analyzed during the current study are available from the corresponding author on reasonable request.

## Abbreviations

A1: 1st assistant
A2: 2nd assistant
AN: Anesthesiologist
AR: Anesthesia room
CAD: Computer-aided design
DES: Discrete Event Simulation
IBCT: Incision-to-begin-of-closure-time
IHT: Instrument handover time
MRI: Magnetic Resonance Imaging
OR: Operating room
OWAS: Ovako Working Posture Analyzing System
R1: Rack 1
R2: Rack 2
SN: Scrub nurse
ST1: Supply stock 1
ST2: Supply stock 2
SU: Surgeon
THA: Total Hip Arthroplasty
TKA: Total Knee Arthroplasty
TRM: Total Rotational Movement
WP1: Working place 1
WP2: Working place 2
