# Supplementary material for: Surgical workflow simulation for the design and assessment of operating room setups in orthopedic surgery
Source: BMC Med Inform Decis Mak. 2020 Jul 2;20:145. doi: 10.1186/s12911-020-1086-3 (PMC7333415; doi:10.1186/s12911-020-1086-3)
Supplement: Supplementary file 1 — Additional file 1 Supplementary file 1. Appendix A – Simulation Implementation. The supplementary data consists of a detailed description of the used software simulation tool (Delmia Quest by Dassault Systèmes) and the implementation of the simulation environment. [file 12911_2020_1086_MOESM1_ESM.docx]

Appendix A – Simulation Implementation

During simulation implementation phase Delmia by Dassault Systèms [60] was used to simulate THA and TKA processes. Delmia provides a 3D Modeling environment and a logical process simulation component. Although Delmia was originally developed for the simulation of manufacturing processes, it has also wide distribution in healthcare (e.g. [7], [46]). Delmia consists of three modeling perspectives. The CAD world enables the creation and import of 3D objects. The Model world allows the graphical implementation of the models and the simulation world contains the simulation and program logic for the process execution of the model.

In the first step, a 3D model of the orthopedic OR including the furnishing was created based on the ORs’ ground plan and on-site measurements of the inventory. Subsequently, the 3D CAD model of the OR is then accessed from the Model world, which enables a graphical implementation of the model. To create a realistic digital OR simulation environment for the evaluation of OR setups, the CAD models were enriched with 3D representations of the OR staff and material resources (e.g. instrument tables and instruments, displays, the OR table as well as medical devices). In the Model world, the graphical representation is based on accurate 3D geometries, which enable the integration of geometric measurements in the simulation and process analysis. For the simulation, the model elements need to be provided with details about their behavior during process executions (e.g. location, activities, speed, process duration). The following classes were implemented in the Delmia simulation, which provides adequate element classes with different functions and configuration options:

• *Parts* are changed during the simulation (e.g. patient, instruments)

• *Source and Buffer* of parts (e.g. instrument tables, shelves)

• *Machines* are processing parts (e.g. medical devices)

• *Labor* transports parts and use machines (e.g. surgeon, scrub nurse, circulator)

• *Accessories* are without function or process logic (e.g. OR table, displays)

The intraoperatively recorded processes of THA and TKA surgeries were modeled in Delmia. Consequently, the model elements were enriched with process logic and the initially recorded process parameters. Thereby, the instrument handover processes were simulated, including the amount, pathway and duration of the handovers between the surgeon and the scrub nurse. In addition, the high-level surgical activities, the incision-to-begin-of-closure time of the surgery and the circulators’ travel path were included in the simulation to evaluate alternative OR layouts in different simulation studies. Finally, the implemented simulation models were executed in Delmia and verified on the base of the recorded intraoperative data.
