# Supplementary material for: Surgical workflow simulation for the design and assessment of operating room setups in orthopedic surgery
Source: BMC Med Inform Decis Mak. 2020 Jul 2;20:145. doi: 10.1186/s12911-020-1086-3 (PMC7333415; doi:10.1186/s12911-020-1086-3)
Supplement: Supplementary file 2 — Additional file 2 Supplementary file 2. Appendix B – Simulation Experiments. The supplementary data presents the conducted simulation experiments in detail. Especially, the simulation of the instrument handover times between scrub nurse and surgeon as well as the simulation of the circulators’ travel path is described. [file 12911_2020_1086_MOESM2_ESM.docx]

Appendix B – Simulation Experiments

*Instrument Handover Times*

During data acquisition, the number and pathway of instrument handovers between surgeon and scrub nurse were recorded in the initial setups (how many instruments are taken from which instrument table). Additionally, the handover time was measured for the handovers from different instrument tables. The recorded instrument handover times were approximately normal-distributed, which was also assumed in the simulation. Based on the intraoperatively recorded handover times a mean handover time and the standard deviation were calculated. During the simulation, the realizations of handover times were sampled from the underlying distribution.

Based on the handover times, time for rotational movement and the distance of the instrument tables a mean handover speed was calculated and verified with the initial intraoperative recordings. For the scenario implementation of the newly designed OR setups, the mean handover speed was used to sample the handover times of the new OR setups. The adequate number of instrument handovers were performed in the simulation environment and the resulting handover time, as well as the total handover time of one surgery, was calculated by Delmia.

*Travel Path of the Circulator*

In every simulation scenario, the travel path of the circulator was calculated and combined with the instrument handover process simulation. During process acquisition, the number and pathways for each way, e.g. to a supply stock or to the scrub nurse for material handover, were recorded. The 3D representation of the simulation environment was used to calculate the distance of different travel paths. In the simulation, the pathways to the different destinations were analyzed considering different possible passages between instrument tables, the OR table and other furniture with a minimum distance to the sterile area. The shortest possible path considering these constraints was defined as the optimal pathway of the circulator. With the help of Delmia, it was possible to model the pathways of the initial and newly designed setups. Thereby, every intraoperatively recorded travel path was simulated, and the total distance was calculated, which was traveled by the circulator during a THA or TKA surgery.
